# Review: Occurrence and Distribution of Galanin in the Physiological and Inflammatory States in the Mammalian Gastrointestinal Tract

**DOI:** 10.3389/fimmu.2020.602070

**Published:** 2021-01-22

**Authors:** Marta Brzozowska, Jarosław Całka

**Affiliations:** Department of Clinical Physiology, Faculty of Veterinary Medicine, University of Warmia and Mazury, Olsztyn, Poland

**Keywords:** galanin, galanin receptors, inflammation, gastrointestinal tract, neuropeptide

## Abstract

Galanin (GAL) is a broad-spectrum peptide that was first identified 37 years ago. GAL, which acts through three specific receptor subtypes, is one of the most important molecules on an ever-growing list of neurotransmitters. Recent studies indicate that this peptide is commonly present in the gastrointestinal (GI) tract and GAL distribution can be seen in the enteric nervous system (ENS). The function of the GAL in the gastrointestinal tract is, *inter alia*, to regulate motility and secretion. It should be noted that the distribution of neuropeptides is largely dependent on the research model, as well as the part of the gastrointestinal tract under study. During the development of digestive disorders, fluctuations in GAL levels were observed. The occurrence of GAL largely depends on the stage of the disease, e.g., in porcine experimental colitis GAL secretion is caused by infection with *Brachyspira hyodysenteriae*. Many authors have suggested that increased GAL presence is related to the involvement of GAL in organ renewal. Additionally, it is tempting to speculate that GAL may be used in the treatment of gastroenteritis. This review aims to present the function of GAL in the mammalian gastrointestinal tract under physiological conditions. In addition, since GAL is undoubtedly involved in the regulation of inflammatory processes, and the aim of this publication is to provide up-to-date knowledge of the distribution of GAL in experimental models of gastrointestinal inflammation, which may help to accurately determine the role of this peptide in inflammatory diseases and its potential future use in the treatment of gastrointestinal disorders.

## Review Methodology

The scientific papers that were reviewed in this article were researched in journal databases as well as specialized topic websites. Terms that were used in article searches included the pathomechanism of GAL, its role in the mammalian digestive system and its contribution to ongoing inflammation. The criteria for inclusion of data for the review required articles to be directly related to the topic of neuropeptide function and to be peer-reviewed. Both qualitative and quantitative articles were reviewed. Quality articles provide insight into the problem and help understand the causes. In contrast, quantitative articles use measurable data to form facts and discover patterns in research.

## Introduction

GAL is a neuropeptide with N-terminal glycine and C-terminal alanine amide. It was first isolated from the sections of the porcine upper small intestine in 1983 (1, 2). The presence of GAL has been demonstrated in both the central and peripheral nervous systems of many mammalian species (1). The action of the peptide focuses on the modulation of physiological functions, including sleep regulation, nociception, and cognition. It has been confirmed that GAL also controls the functioning of the neuroendocrine system by affecting feeding, thermoregulation, cellular energy metabolism as well as osmotic and water balance (3–5).

In the mammalian gastrointestinal tract, GAL is described in the enteric neurons in both the submucosal (SP) and myenteric plexuses (MP) (6). It was noted that GAL immunoreactive nerve fibers are present in the mucous layer as well as in the muscle layers (7). The effect of GAL on the digestive system is multiple. It is responsible for inhibiting the secretion of active substances such as somatostatin, insulin, and glucose (1, 8). GAL inhibits gastric acid secretion and is also involved in the regulation of gastric and intestinal motility. GAL stimulates and inhibits gastrointestinal transit and acts directly on smooth muscle cells or indirectly through activation internal neural pathways (1). Due to the large number of described physiological functions of GAL in the gastrointestinal tract, there is a growing interest in the role of GAL in the development of gastrointestinal diseases.

The following sections provide a brief overview of GAL activity in the mammalian digestive tract, both in the physiological state and as a result of gastrointestinal pathology. The data collected in this review are necessary to provide an accurate understanding of the role of GAL in the digestive system of many animal species. The overview includes information on this peptide’s role during ongoing inflammation. Due to the incoming reports on the protective effect of GAL, the collection of the latest data indicating the involvement of the GAL in the pathomechanism of the development of digestive disorders will be helpful for researchers in the field of gastroenterology, pharmacology, and neurology (9).

The galanin system is widely involved in neuromodulation and neurotransmission. GAL is the main signaling molecule in the galaninergic system. The GAL neuropeptide consists of 29 amino acids and contains a C-terminal amidated glycine. In humans, GAL is composed of 30 amino acids and contains a C-terminal non-amidated serine (3).

**Figure 1:**
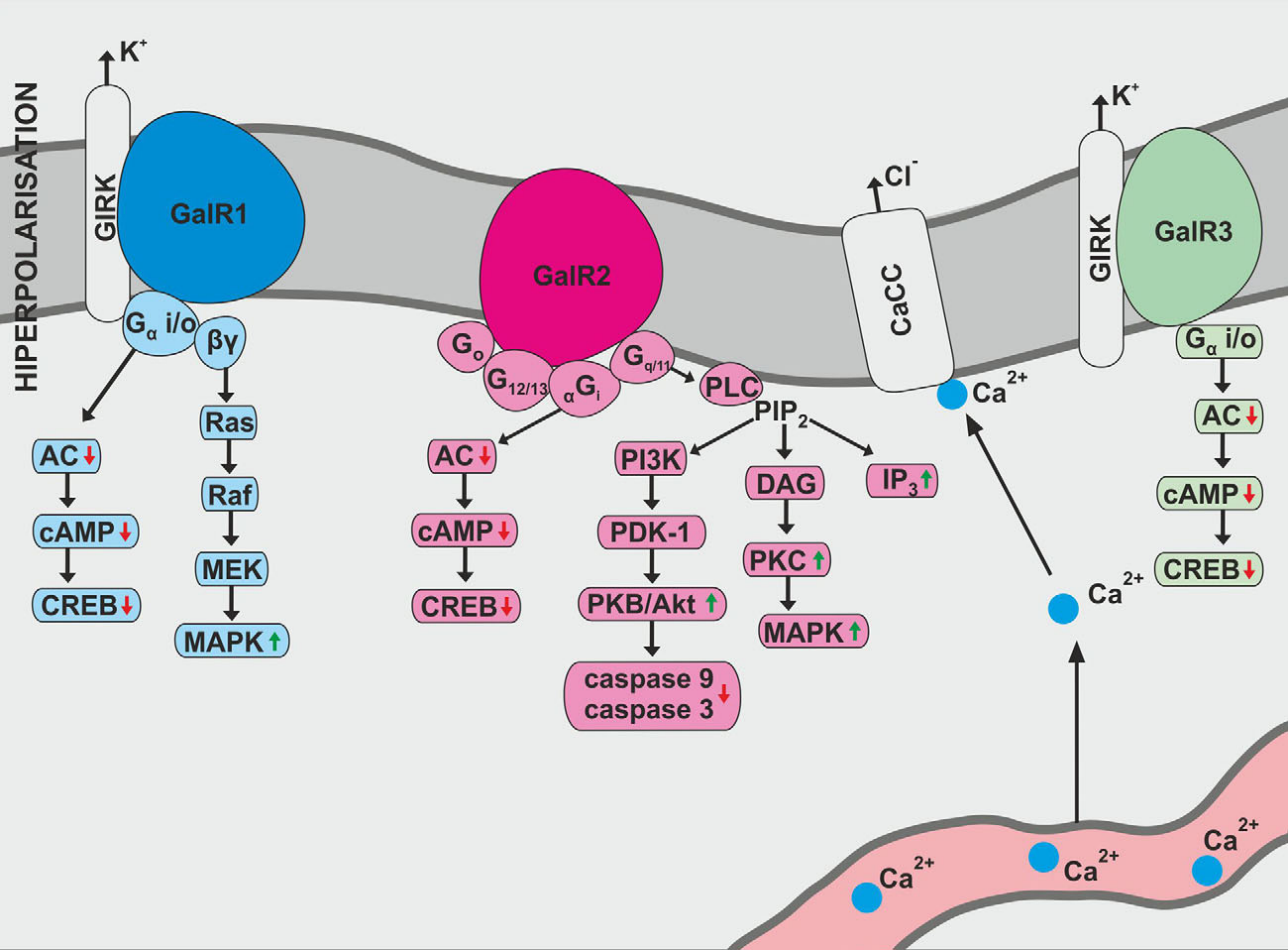
Biological action of galanin receptors—GalR1, GalR2, and GalR3. AC, adenylate cyclase; CaCC, Ca^2+^-dependent chloride channel; cAMP, 3′,5′-cyclic adenosine monophosphate; CREB, cAMP response element binding protein; DAG, diacylglycerol; IP3, inositol triphosphate; MEK, mitogen-induced extracellular kinase; PDK-1, phosphoinosotide-dependent protein-kinase 1; PIP2, phosphatidylinositol bisphosphate; PI3K, phosphatidylinositol 3-kinase; PKB, protein kinase B; PLC, phospholipase C.

GAL exhibits a variety of biological effects due to three known galanin receptor subtypes: GAL1, GAL2, and GAL3 (**Figure 1**). All galanin receptor subtypes are members of the G protein-coupled receptor (GPCR) family (10). The receptor subtypes cause variable signaling activities, which translates into various physiological effects of galanin. In addition, receptor function may be slightly changed in different cell populations (10).

GAL1 was the first galanin receptor described and has been discovered in the human melanoma cells (11). The human GAL1 protein, consisting of 349 amino acids, is encoded by the GAL1 gene located on chromosome 18q23  (12). In the rat, GAL1 contains only 346 amino acids and has 92% similarity with human galanin receptor type 1 (10).

According to Habert-Ortoli et al., induction of rat or human GAL1 expressed in transfected cell lines blocks forskolin-stimulated cAMP synthesis in a pertussis toxin (PTX)-sensitive manner (11). Receptor biological function is associated with adenylate cyclase activity and cAMP production (1). In addition, GAL1 activation opens the G protein-coupled inwardly-rectifying potassium channels (GIRKs) and stimulates MAPK (mitogen-activated protein kinase) independently of protein kinase C, which is susceptible to PTX inhibition (1, 13). Studies also confirm GAL1 activation at the cellular level induced prolonged activation of extracellular signal-regulated kinase 1 and 2 (ERK 1 and 2) through Gαi-subunits (10). This action consequently leads to a modification of the cyclin-dependent kinase inhibitor 1B and 1C (p27^Kip1^ and p57^Kip2^) expression and inhibition of cell proliferation (1, 10).

Galanin receptor type 2 was originally isolated as expressed cDNA from rat tissue (14). The human receptor consists of 387 amino acids (10). GAL2 activation occurs through a variety of classes of G-proteins and is associated with the stimulation of many intracellular pathways. The most described pathway involves phospholipase C activation (15). Inositol phosphate hydrolysis is intensified, mediating Ca^2+^ release into the cytoplasm from intracellular storage and opening Ca^2+^ -dependent chloride channels (1).

There is also evidence that the interactions between GAL2 and G_i_-type G-proteins are quite ambiguous. Rat GAL2 transfected into Chinese hamster ovary (CHO) cells and human embryonic kidney 293 (HEK-293) cells did not alter forskolin-stimulated cAMP accumulation after galanin activity (14, 15). Galanin-dependent blocking of forskolin-stimulated cAMP production was noted in CHO cells transfected with rat GAL2 (14). The same relationship can be noted in HEK-293 cells population (15).

Interestingly, activation of GAL1, as well as GAL2, inhibits the cyclic AMP-responsive element-binding factor (16). It has also been reported that GAL2 signaling pathways react with G_o_-type G-protein, resulting in activation of MAPK protein (1). GAL2 has been shown to be involved in neuronal survival and apoptosis. It is also associated with the PI3K-Akt pathway causing inhibition of caspase-3 and caspase-9 activity (17).

Galanin receptor type 3, originally isolated from rat tissue, encodes for a protein consisting of 370 amino acids (10). It was also noted that similarity between rat GAL3 and GAL1 molecules was 36%, and for GAL3 and GAL2 receptors it was 55% (10). In turn, human GAL3 contains 368 amino acids and has 90% similarity to the amino acid sequence of rat GAL3 protein (1).

The GAL3 signaling properties are still unclear. GALR3 activity has been described as combining the effects of GAL1 and GAL2 signaling (1). Other studies have revealed that cloned GAL3 react with G_i/o_-type G-protein, which causes PTX-sensitive stimulation of the internal K^+^ current during simultaneous expression with GIRK1 and GIRK4 in Xenopus oocytes (18).

## Distribution and Biological Function of Galanin in the Gastrointestinal Tract

**Figure 2:**
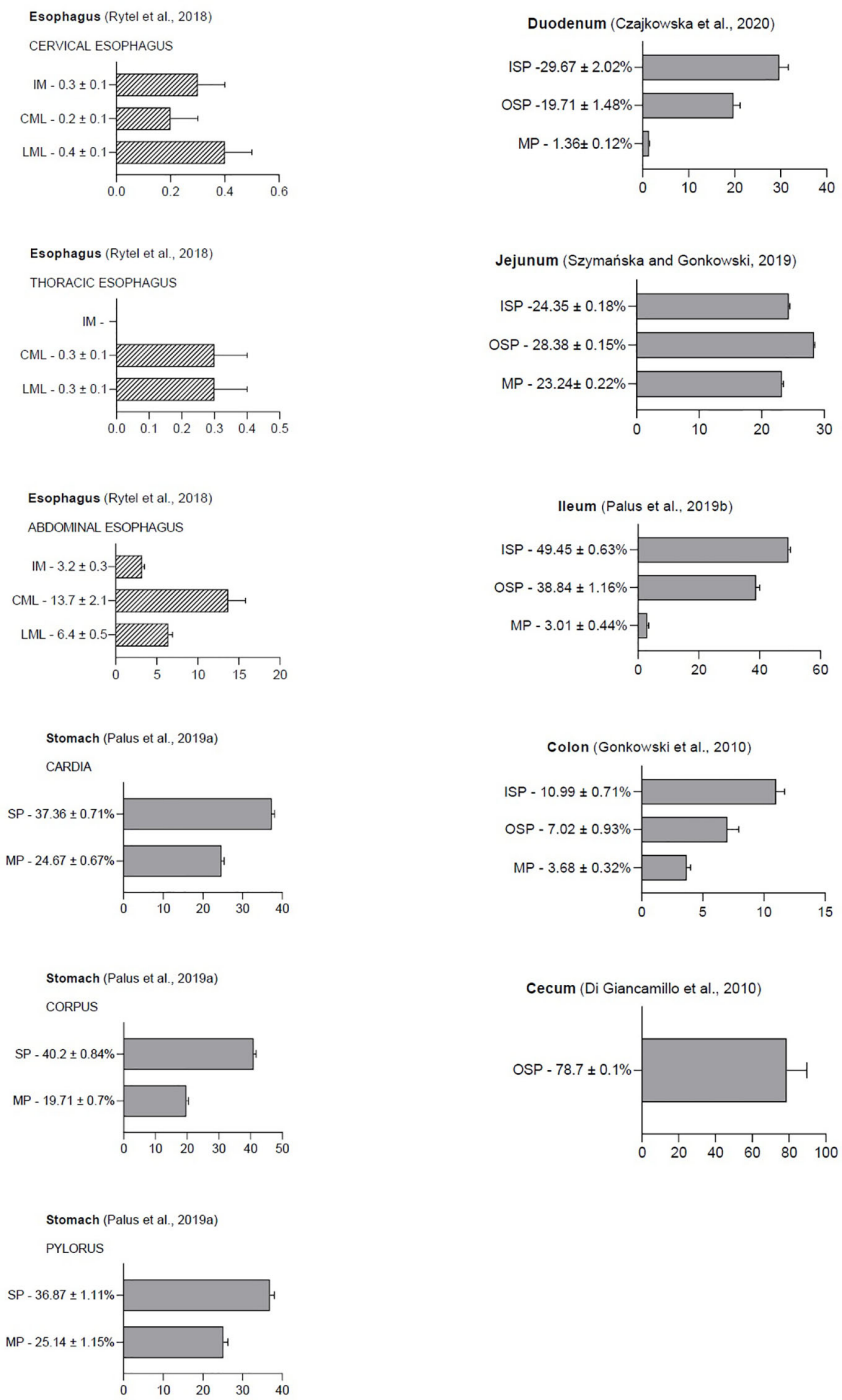
The following graphic displays the result diagrams of the physiological distribution of galanin in the porcine gastrointestinal tract visualized using immunofluorescence staining. Esophageal data (dashed bars) are expressed as the number of GAL-positive nerve fibers in the longitudinal muscle layer (LML), circular muscle layer (CML), and intestinal mucosa (IM). In the stomach, duodenum, jejunum, ileum, colon, and rectum, the results are presented as the percentage of GAL-IR neurons (grey bars) in the myenteric plexus (MP), inner (ISP), and outer submucosal plexus (OSP). The percentage of neurons was determined by counting cells showing co-localization of two peptides—PGP 9.5 (a pan neuronal marker) and GAL—in relation to the total number of neurons in a given population.

Because GAL was first isolated from the porcine small intestine, research is still ongoing into the possible peptide function in the gastrointestinal tract in various species (2). It has been reported that GAL is widely distributed in the GI and GAL occurrence is present in enteric structures in many mammalian species, such as pigs, dogs, rats, and guinea pigs (6, 19, 20). The physiological levels of GAL in the porcine gastrointestinal tract are shown in **Figure 2**.

GAL has many biological effects in the digestive system. GAL is responsible for inhibiting the secretion of gastric acid and pancreatic peptides such as insulin, glucagon, and somatostatin (21–24). In addition, GAL regulates gastrointestinal motility directly by acting on gastrointestinal smooth muscle cells or indirectly by neuromodulation and stimulating the synthesis of other factors (25, 26). The effects of GAL on the functioning of the gastrointestinal tract are largely determined by the species as well as the fragment of the digestive tract studied.

The role of GAL in gastrointestinal pathology is also still of interest. This peptide has gained recognition due to its abundant distribution in the enteric nervous structures and its activity in the digestive tract (27). It is widely known that neuromessengers synthesized by enteric neurons are involved in the development of gastrointestinal disorders. Many reports have indicated that communication between the enteric nerves *via* neurohormones and GAL plays an important role in the pathogenesis of gastrointestinal inflammation (27).

## Esophagus

Immunoreactivity to GAL was observed in the esophagus in many species such as pigs, opossums, rats, guinea pigs, mice, and humans (6, 20, 28, 29). Studies using the immunofluorescence staining technique revealed that only single GAL-positive cells were visible within the muscular esophageal myenteric plexuses of the rat and mouse (6). However, the presence of GAL-IR neurons in the submucosal plexus was not observed. It should be noted that the latter plexus is very rare in the esophagus (6). Very few fibers staining for GAL in the rat and mouse were found in esophagus. In pigs, the number of GAL-positive nerve fibers was the highest in the abdominal esophagus, particularly in the circular muscle layer (20). It is proven that in this part of the gastrointestinal tract, GAL is involved in regulatory processes associated with esophageal peristalsis (30). It inhibits motor endplates in esophageal striated muscles. In addition, there have been reports that GAL may reduce mechanosensitivity in the vagal nerves supplying this part of the GI (31).

Cheng et al. studies on rat models with adriamycin-induced esophageal atresia revealed an increase in the IR density of GAL in the experimental group (32). The increased GAL level found in this study suggests reduced peristalsis and relaxation failure of the lower esophageal sphincter. This image is similar to organ achalasia (32). In the esophagus of the opossum, especially in the abdominal region, GAL is less amplified by peristaltic contraction (28). It is thought that GAL may selectively affect the noncholinergic component responsible for esophageal peristalsis (28). Unfortunately, the mechanism of this blocking has not been explained. **Table 1** provides information on GAL plasticity in the stomach and esophagus under the influence of inflammation in various animal species.

**Table 1:** Distribution of galanin in esophagus and stomach in inflammatory conditions.

Organ	Species	Galanin regulation	Experimental model	References
***Esophagus***				
Abdominal region	*opossum*	Galanin-IR increased	Experimentally adriamycin-induced atresia	(32)
***Stomach***				
Prepyloric region	*pig*	Number of galanin-IR neurons increased	Experimentally induced hyperacidity	(33)
Cardia, corpus, pylorus	*pig*	Number of galanin-IR neurons increased	Acrylamide supplementation	(34)
Antrum, pylorus	*pig*	Number of galanin-IR neurons increased	Experimentally induced diabetes	(35)
Antrum	*mouse*	Galanin concentrations decreased (RIA)	Experimentally induced diabetes	(36)
Antrum, pylorus	*pig*	Increased in number of galanin-IR neurons Exacerbation of mRNA encoding galanin and GAL1	Experimentally induced ulcerations	(37)
Fundus	*pig*	Number of galanin-IR nerve fibers and neurons increased	Intragastric *Brachyspira hyodysenteriae* administration	(38)

## Stomach

In the stomach, GAL is commonly found in both the myenteric and submucosal plexuses in all parts of the organ (34). The presence of GAL has been confirmed in studies conducted on many species such as pigs, mice, rats, dogs, guinea pigs, and humans (34, 39, 40). Interestingly, significantly more GAL-positive neurons occur in the gastric submucosal plexuses, which are primarily responsible for regulating the gastrointestinal secretory functions (41). However, although extensive fiber systems staining for GAL were observed in the stomach in the rat, mouse, and pig, only a few GAL-IR fibers were noted in guinea pigs (6). Recent studies indicate that GAL affects gastric emptying and inhibits gastric secretion (37). It is well known that acetylcholine, somatostatin, histamine, gastrin and many other neurohumoral substances are involved in gastric acid secretion (42, 43). The inhibition of gastric acid secretion by GAL is stimulus-specific because it only inhibits the response to gastrin as well as gastrin-releasing substances such as neuromedin C (43). GAL infusion does not affect gastric acid secretion by cholinergic agent or histamine (43). Research suggests that somatostatin may not play a significant role in the inhibitory effect of GAL on gastric acid secretion, because GAL causes a reduction in the somatostatin level in the stomach and pancreas (41) and is also involved in mucosal epithelial cell absorption and ion transport (44, 45).

The localization of gastric GAL1, GAL2, and GAL3 proteins has also been noted, although the level of GAL2 mRNA was most abundant in the stomach (46). GALRs are closely related to the modulation of gastric contractility as a result of GAL action. GAL has been found to inhibit, and then, stimulate motor activity in the rat stomach, which is the result of stimulation of the GAL1-dependent pathway (46). In contrast, *in vitro* studies have shown that GAL has only a stimulant effect, which suggests a non-neuronal, direct effect on smooth muscles. This action is probably mediated by GAL2 (46, 47). Studies also suggest that GAL may play a role in neuromodulation and neurotransmission (33). It is well known that neuroactive substances secreted from the same neurons can perform similar functions. Recent studies show that in the porcine stomach, GAL co-localizes with vasoactive intestinal peptide (VIP), neuronal nitric oxide synthase (nNOS) and cocaine- and amphetamine-regulated transcript peptide (CART), which may suggest that GAL has a protective effect on cells as well as being involved in regulating gastric blood flow (34).

Many available reports indicate the effect of gastric disorders on GAL presence in ENS. Intragastric supplementation of hydrochloric acid caused an increase in the GAL level in the porcine prepyloric region (33). Administration of acrylamide in pigs resulted in a percentage increase in galaninergic neurons in the cardia, corpus, and pylorus in both enteric plexuses (34). In porcine diabetes models, an increase in GAL-positive cells was observed after streptozotocin supplementation in antrum and pylorus but only in the myenteric plexus. In the submucosal plexuses, the changes were not statistically significant (35). However, studies conducted on the diabetic non-obese mice model have revealed a significant decrease in GAL concentration in the antrum (36). The authors suggest that GAL levels decrease in the early stage of diabetes and increase in the later phase which is associated with organ regeneration (35, 36). Overall and local levels of GAL and GAL1 expression were down-regulated in patients with gastric cancer (48). In induced porcine gastric ulcerations, an increase in GAL level in enteric plexuses was seen (37). The authors demonstrated that acetic acid administration resulted in an increase in GAL-IR neurons as well as a statistical significant increase in expression of mRNA encoding GAL and GAL1 (37). In the rat, a single cysteamine administration caused ulcers to appear in the gastric fundus area. This, consequently, resulted in changes (not statistically significant) in GAL-like immunoreactivity (49). In the case of intragastric administration of *Brachyspira hyodysenteriae*, an increase in the number of enteric GAL-IR nerve fibers and neurons in the examined porcine stomach was noted (38). However, studies aimed at comparing the density of GAL-positive nerve fibers in the control group and in people with gastric adenocarcinoma revealed an increase in GAL-IR nerve fibers in a circular muscle layer and lamina muscularis mucosae in the disease group (40).

## Small Intestine

GAL distribution is present in all parts of the small intestine in many mammalian species such as dogs, pigs, rats, mice, and humans (6, 41, 50, 51). In addition, the results of an immunocytochemical study indicate that GAL occurs in all layers of the porcine, human and rat small intestine, as well as in enteric plexuses (52). It has also been found that for guinea pig preparations, there is a visible distal increase in both GAL-IR fiber density and fluorescence intensity in the small intestine. Low levels of GAL were seen in the duodenum, while it was significantly higher in the ileum (6). Studies to determine the concentration of GAL in individual sections of the small intestine have shown that the level of GAL increased caudally (6). Particularly high GAL content was noted in the porcine ileum (52). In addition, it is well known that GALRs mRNAs are located in the small intestine (53). In studies performed on rat preparations, it was revealed that GAL1 is most commonly found in the ileum. Similar observations were noted for GAL2. The highest level of GAL3 was recorded in the jejunum (46).

The latest research focuses on the role of GAL in inhibiting intestinal peristalsis. GAL has an inhibitory effect on cholinergic and tachykinergic transmission (53, 54). Furthermore, GAL co-localization with nNOS as well as VIP, major neurotransmitters of descending neurons, has also been demonstrated (19). Studies conducted using of guinea pig ileum tissue revealed that GAL1 mediates the cholinergic transmission and intestinal peristalsis activity (54). Moreover, GAL acts on peristalsis by decreasing its efficiency (reduction of peak pressure as well as longitudinal muscle contraction) and reducing the intestinal wall compliance by activating GAL1 (54). GAL has also been shown to inhibit depolarization-induced an increase in the Ca^2+^ concentrations in cultured myenteric neurons. GAL1 is responsible for this effect, which mediates the inhibition of Ca^2+^ influx through voltage-dependent Ca^2+^ channels (53).

It is well known that most of the small intestine neuroendocrine peptides are synthesized in the duodenum (50). Many studies are aimed at proving that pathological conditions in the duodenum significantly affect GAL levels, and this peptide is involved in the development of damage and/or their repair. **Table 2** shows the regulation of GAL in the small intestine during various inflammatory conditions. Acrylamide supplementation in pigs resulted in GAL level increases in all enteric plexuses at both high (5 µg/kg b.w./day) and low (0.5 µg/kg b.w./day) doses per 28 days (7). The same observations were noted during long-term treatment with naproxen in pigs (50). Additionally, Strom et al. have proven that the estradiol treatment of ovariectomized rats resulted in higher levels of immunoreactive galanin in the gut, except in the jejunum (8, 67). In chronic alcoholism in humans, an increase in the density of GAL-positive fibers has been noted, especially in the submucosa of the distal duodenum (56). While there was a clear increase in the density of galaninergic fibers, the results were not statistically significant; most likely due to limited sample size. On the other hand, the proportion of GAL-IR nerve fibers is significantly decreased in the duodenum of type 2 diabetic mice (68). Since GAL is responsible for inhibiting intestinal motility, the authors suggest that it may be involved in peristalsis changes that are observed in people who abuse alcohol (56). Bisphenol A administration increased GAL level in porcine myenteric neurons, but decreased GAL distribution in submucosal plexuses (57). However, in NOD mouse, an animal model of human diabetes type 1, a decrease in GAL concentration in the duodenum was observed (55).

**Table 2:** Presence of galanin in small intestine during various inflammatory conditions.

Organ	Species	Galanin regulation	Experimental model	References
***Duodenum***				
	*Mouse*	Galanin concentration decreased (RIA)	Experimentally induced diabetes	(55)
	*Pig*	Number of galanin-IR neurons increased	Experimentally naproxen-induced inflammation	(50)
	*Human*	Density of GAL-IR nerve fibers increased in submucosa	Chronic alcoholism	(56)
	*Pig*	Number of galanin-IR neurons increased in MP and decreased in SP	Bisphenol A supplementation	(57)
***Jejunum***				
	*Pig*	Number of galanin-IR neurons increased in SP and decreased in MP	Supplementation with red kidney bean (*Phaseolus vulgaris*) lectin	(58)
	*Pig*	Number of galanin-IR neurons increased	Bisphenol A supplementation	(59)
	*Rat*	No changes of galanin levels (IHC)	Syngeneic small bowel transplantation	(60)
***Ileum***				
	*Pig*	Number of galanin-IR neurons increased	Proliferative enteropathy	(61)
	*Pig*	Density of GAL-IR nerve fibers and galanin concentration increased	Intestinal *Brachyspira hyodysenteriae* infection	(62)
	*Rat*	Submucosal and myenteric neurones expressing galanin mRNA increased Number of galanin-IR neurons increased	Experimentally induced ileal hypertrophy	(63)
	*Pig*	Density of GAL-IR nerve fibers decreased in circular muscle layer	ZEN supplementation	(64)
	*Pig*	Number of galanin-IR neurons increased	Acrylamide supplementation	(65)
	*Rat*	No changes of galanin expression (IHC)	Buserelin-induced enteric neuropathy	(66)

According to research by Zacharko-Siembida et al., supplementation with red kidney bean (*Phaseolus vulgaris*) lectins resulted in a statistically significant increase in GAL distribution in submucosal neurons (79.1 ± 5.3% in experimental vs. 56.8 ± 6.4% in control) in porcine jejunum. Changes in the enteric cells of the myenteric plexuses were not observed (58). Lectins have been shown to interfere with several aspects of intestinal physiology, including absorption and secretion (69). It is not surprising that their administration caused changes in GAL level, which is involved in the regulation of intestinal secretion. However, the administration of bisphenol A at both low and high doses revealed an increase in the number of GAL-positive neurons in all enteric ganglia in the analogous segment of the intestine (59). The authors suggest that the increase in GAL presence in the porcine jejunum is closely related to the neuroprotective properties of the peptide. Syngeneic small bowel transplants in a rat, aimed at demonstrating changes in the functioning of the jejunal enteric nerves without the effect of the immune system, revealed that the density and distribution of GAL-IR nerve fibers did not change within 10 days, 100-day, or 400-day isografts (60). This may suggest that immunological factors affect GAL level in diseased tissues.

Enteric neurons are known to show a high degree of plasticity in response to inflammation. In the porcine ileum, *Lawsonia intracellularis* infection caused proliferative enteropathy, which resulted in changes in GAL distribution (61). Immunofluorescence staining also showed an increase in the number of GAL-containing neurons in each enteric plexus (61). The same observations were noted in the ileum of a pig after *Brachyspira hyodysenteriae* infection (62). The study showed that in the experimental group, the GAL concentration in the mucosa was significantly higher. GAL-positive nerve fibers were mainly found in the interfollicular regions of Peyer’s patches in the mucosa and intestinal villi and as well as in the dome areas of the follicles. These are regions where T lymphocyte subpopulations (CD2^+^, CD4^+^, CD5^+^, CD8^+^, TCR-γ^+^) can be found (62). This suggests that GAL may be involved in T-cell function in the ileum. It is assumed that GAL is involved in the functioning and interaction between the neurological and immune systems (62). This peptide probably acts through GAL1 and GAL3, exerting antiproliferative and proapoptotic effects (70). A significant increase in GAL level was observed in rat hypertrophic ileum compared to the control tissue (63). GAL immunoreactive nerve cells in myenteric plexuses increased threefold while GAL mRNA expressing neurons showed an almost sevenfold increase. By *in situ* hybridization, the percentage of submucosal neurons expressing GAL mRNA increased from 8% in the control to 22% after obstruction (63). The increase in GAL level in this part of the intestine was also caused by the oral administration of acrylamide (a potential carcinogenic compound formed in food products subjected to high temperature) in pigs (65). However, the administration of low doses of zearalenone (ZEN)—an estrogenic mycotoxin—caused a decrease in the amount of GAL immunoreactive nerve fibers within the circular muscle layer of porcine ileum (64). Because the expression of protective neuromessengers, including GAL, increases during most pathological stimuli, it can be assumed that low doses of ZEN did not trigger the protective mechanisms in the pig ileum (64). Interestingly, the buserelin treatment (model of enteric neuropathy) in rats did not cause changes in the presence of GAL or other neurotransmitters (66). In contrast, the authors noted a significant loss of enteric neurons. These observations suggest that neuronal loss is not selective. ENS has adaptive properties and strives to preserve its original set of neuronal subpopulations as well as its distribution and density of nerve fibers (66).

## Large Intestine

GAL distribution is noticeable in the large intestine. There are many studies in the literature describing the role of GAL in the colon, while the GAL function in the cecum and rectum is almost unknown.

The presence of GAL-positive colon nervous structures is seen in rats, mice, pigs, guinea pigs and humans (5, 6, 71–73). Studies show that GAL level is significantly higher in submucosal neurons than in myenteric neurons (73). In pigs, the number of GAL-positive neurons amounts to 11.20% in the colon myenteric plexuses, while in the inner submucosal plexuses 4.03% of GAL-immunoreactive neurons have been shown (73). Similar changes were seen in the guinea pig colon (6). GAL-positive nerve fibers, often innervating walls of blood vessels, were found in greater quantity in the submucosal layer in the pig and guinea pig (6). Studies have revealed that a much higher density of GAL-immunoreactive nerve fibers was found in the colon circular muscle layer, whereas only a few single fibers could be detected in the longitudinal muscle layer in pigs, rats, guinea pigs and mice (6, 73). According to the results obtained by Anselmi et al., all three types of GAL receptors are found in the rat colon. Interestingly, a particularly high level of GAL1s mRNA was described in this part of large intestine (46). Since GAL1 is particularly involved in intestinal peristalsis, it is suggested that in the colon it is also largely responsible for GAL binding and affects intestinal contractility (46). In the human colonic epithelial cell line T84, GAL1 was the only expressed GALRs and its activation caused chloride secretion (74). In addition, based on the observations that the nuclear factor κB (NF-κB) increases GAL1 expression, it has been shown that this receptor is involved in gastrointestinal inflammation (74). It is also suggested that GAL and GAL1 are important mediators of the colonic fluid secretion in diarrhea caused by various intestinal pathogens (75).

Colitis is inflammation of the large intestine, which is characterized by multiple etiology. Each inflammatory factor causes a unique pattern of pathological development, which include changes in neuromessenger expression, including GAL. **Table 3** shows the plasticity of GAL in the large intestine in various experimental models. In a study to determine the change in GAL distribution as a result of inflammation caused by long-term administration of trinitrobenzene sulfonic acid, an increase in GAL immunoreactivity was observed (77). Human diverticulitis in the colon also revealed an increase in GAL level as a result of the development of inflammation (79). These observations are similar to the results of Gonkowski et al. studies performed on the porcine model (73). Formalin injection- and axotomy-induced colitis showed an increase in GAL-positive enteric neurons and nerve fibers (73). Interestingly, Matkowskyj et al. showed that infection with *Salmonella typhimurium* causes an increase in the immunoreactivity of GAL and GAL1 in the enteric nervous system (78). These changes are closely associated with increased NF-kB expression, which may suggest its involvement in the GAL synthesis in the ENS (78). It is also significant that an increased level of GAL1-immunoreactivity was observed in the dextran sulfate sodium model of murine colitis (80).

**Table 3:** Distribution of galanin in large intestine in various animal experimental model.

Organ	Species	Galanin regulation	Experimental model	References
***Cecum***				
	*Pig*	No changes of galanin expression (IHC)	Dietary supplementation with *Pediococcus acidilactici*	(76)
***Colon***				
	*Rat*	Galanin-LI increased in mucosa and muscular layer (IHC)	Experimentally induced colitis (administration of trinitrobenzene sulfonic acid)	(77)
	*Pig*	Number of galanin-IR nerve fibers and neurons increased	Formalin injection- and axotomy-induced colitis	(73)
	*Mouse*	Galanin-LI and GAL1 level increased (IHC)	Infection with *Salmonella typhimurium*	(78)
	*Human*	GAL-LI increased (IHC)	Chronic diverticulitis	(79)
	*Mouse*	GAL1-immunoreactivity increased	Dextran sulfate sodium-induced colitis	(80)
	*Human*	Galanin level increased (PCR)	Colon adenocarcinoma	(81)
***Rectum***				
	*Human*	GAL level and number of galanin-IR myenteric neurons decreased	Colorectal carcinoma	(71)
	*Human*	GAL-LI increased (IHC)	Human immunodeficiency virus infection	(82)

Recent research suggests that GAL may be considered a biomarker of colon cancer. In studies aimed at determining serum GAL levels in patients with colon cancer compared to serum from non-cancerous controls revealed a significant increase in its level in the case of colon adenocarcinoma (81). The GAL level in healthy patients was 25.6 ± 14.5 ng/ml, while in those with colon cancer, it was significantly higher (41.4 ± 19.0 ng/ml) (81). Additionally, GAL overexpression was noted in all colon adenocarcinoma cells tested (LOVO, HCT116, SW480, and SW620 cells), but not in the A549 lung cancer cell line, OVCAR3 and SKOV3 ovarian cancer cell lines, or the HS1 testicular cancer cell line (81). Because GAL inhibits cell proliferation, an increase in its synthesis may be associated with the activation of protective mechanisms in patients with colon adenocarcinoma (81, 83).

However, Godlewski and Pidsudko noted a decrease in GAL levels in colorectal carcinoma tissues compared to the control group, as well as an increase in GAL presence within MP neurons (71). This may suggest a possible role for GALergic innervation in the development of the clinical symptoms reported by patients suffering from colorectal cancer (71, 84). The most frequent reported adverse complaints are alternating constipation and diarrhea. The exact mechanism of these effects is not well known, but it may result in an increase in the contractile activity of the cancer-infiltrated section of the intestine, caused by altered (and partially damaged) innervation of the colon wall, causing disturbances in intestinal peristalsis (71, 84).

The presence of GAL has been demonstrated in the cecum. Studies have revealed that a significant population of GAL-positive cells showed significant co-localization with choline acetyltransferase (ChAT)-IR neurons (76). Since acetylcholine is necessary to maintain intestinal motility, a similar role of GAL in this part of the intestine can be assumed (85). In studies carried out on mouse and rat preparations, GAL-positive nerve fibers were found in the caecum, particularly well visualized in the enteric plexuses (6). A significant number of GAL-IR nerve fibers have been reported in the circular muscle layer of the human caecum (86). A small population of these structures co-expressed with CART. Research suggests that CART is a neuromodulator in the digestive tract, and is involved in contractility and neuroprotection (87, 88). It is widely accepted that the co-location of two neurotransmitters is associated with similar biological effects in the caecal enteric nervous system.

Knowledge of changes in GAL distribution due to pathological changes in the cecum is limited. In pigs, dietary treatment with *Pediococcus acidilactici* was not associated with significant quantitative changes in GAL-positive neurons and glial cells in the cecum (76). Double immunofluorescence, used to identify the nature of neurons, revealed that 100% of GAL-IR positive neurons exhibit positive staining against ChAT in submucosal plexuses. This dependence only applies to GAL-positive cells vs ChAT-IR neurons, but not vice versa (76). The GAL function in this segment of the intestine, as well as its interaction as a result of dysfunction, still needs to be determined.

In the rectal area, GAL presence has been found in humans, pigs, mice, rats, guinea pigs and calves both in enteric plexuses and in nerve fibers (6, 71, 89). In bovine preparations, neurons containing both GAL and VIP were located near the glands and blood vessels. Most likely, these cells perform vaso- and secretomotor functions (89). This is in line with reports describing similar neurons in pigs and guinea pigs (90, 91).

There is very little information in the available literature regarding changes in GAL levels during pathological conditions in the rectum. In rectal biopsy samples of human immunodeficiency virus (HIV)-seropositive and HIV-seronegative patients, increased GAL immunoreactivity in the muscularis mucosa was observed in the study group, but these changes were not statistically significant (82).

## Regenerative Effect of Galanin in the Gastrointestinal Tract

GAL has numerous pleiotropic biological effects, including involvement in protective and regenerative processes in the gastrointestinal tract. Yamaguchi et al. demonstrated that GAL promotes mucosal‐type mast cell (MMCs) differentiation *in vivo*. The authors suggest that GAL that is released from the submucosal plexus may contribute to the proliferation and differentiation of MMCs during ongoing enteritis (92). In a rat model of experimentally induced acute colitis *via* 2,4,6-trinitrobenzenesulfonic acid (TNBS), GAL administration resulted in a reduction in macroscopic damage in the colonic mucosa (93). The authors also noted reduced myeloperoxidase activity and a reduction in the degree of polymorphonuclear neutrophil infiltration, as well as a decreased TNF-α levels and expression of inducible nitric oxide synthase (93). The anti-inflammatory effect of GAL has been demonstrated in chronic TNBS-induced colitis. In another study on acute colitis, the reduction in myeloperoxidase activity and TNF-α levels was less pronounced (9). This suggests that the anti-inflammatory effect of GAL is enhanced in the acute stage of the disease.

As a result of GAL administration, diarrhea decreased in acute TNBS-induced colitis in the rat (9). However, the research of Marrero et al. revealed that in dextran sulfate-induced colitis in mice, GAL supplementation led to increased fluid secretion (80). These differences may be due to other routes of GAL administration as well as to the dosing schedule. However, in both *Salmonella* infection and *Rhesus rotavirus* infection, GAL treatment increased fluid secretion (75, 94). The same observations were noted in cultured human colon epithelial cells exposed to pathogenic *E. coli* (95). It has been proven that diarrhea associated with fluid secretion disorder does not occur in GAL1 knockout mice and that this disorder is of no importance for the development of the inflammatory response following microbial infection (75, 94). According to Matkowskyj et al., an increase in myeloperoxidase activity in the colon of GAL1 knockout mice after experimentally induced *Salmonella* infection suggests that GAL1 mediates the anti-inflammatory GAL response in innate immunity in the colon (75).

GAL treatment may affect the volume and mass of cancerous tumors (96). In the rat model, GAL injection in gastric or colon cancer resulted in a significant inhibition of carcinogenicity (96, 97). Interestingly, the number of blood vessels was clearly reduced in mice receiving continuous intraperitoneal GAL infusion compared to the control group (97). In vitro GAL stimulation of tumors resulted in a reduction in viable cells and the proliferation rate (98). Because GAL inhibits cell proliferation, low GAL level may promote cancer growth and lymph node metastasis. Kozłowska et al. proved that the mechanism of gastric myenteric plexuses degradation in cancer patients was increased, which was correlated with an increased expression of CASP3 or CASP8 (99). These changes were accompanied by a decrease in GAL immunoreactivity (100). It is suggested that reduced GAL levels may be a marker of gastric carcinogenicity in the future.

## Conclusion

The current literature indicates that GAL plays an essential role in many physiological functions of the digestive tract. However, little information is available on the effects of GAL and its receptors on the esophagus and the large intestine. GAL is undoubtedly involved in the regulation of inflammatory processes through neuronal mechanisms or direct receptor-mediated cellular effects. Galanin may also have a direct non-receptor-mediated action on cells to alter the cellular expression levels of other peptides.

The importance of GAL as an inflammatory modulator in gastrointestinal tract is confirmed by data obtained from several experimental models for the study of inflammation. Numerous studies and observations irrefutably point to the fact that GAL suppresses the inflammatory response by regulating various mechanisms of innate immunity, such as the production of pro-inflammatory cytokines. It seems therefore attractive to speculate that the reaction to an exacerbated inflammatory response is an increase in GAL expression in order to restore homeostasis. Understanding the mechanism of GAL action may allow the development of new therapeutic agents or the identification of drug targets to treat inflammatory diseases.

## Author Contributions

MB and JC contributed to the preparation, revision, and approval of the final manuscript. All authors contributed to the article and approved the submitted version.

## Funding

This project was financially co-supported by the Minister of Science and Higher Education in the framework of the program entitled “Regional initiative of Excellence” for the years 2019 to 2022, project no. 010/RID/2018/19, amount of funding is 12,000,000 PLN.

## Conflict of Interest

The authors declare that the research was conducted in the absence of any commercial or financial relationships that could be construed as a potential conflict of interest.
